# The impact of the COVID-19 pandemic and the expansion of free vaccination policy on influenza vaccination coverage: An analysis of vaccination behavior in South Korea

**DOI:** 10.1371/journal.pone.0281812

**Published:** 2023-02-15

**Authors:** Muhan Yeo, Jeongmin Seo, Juwon Lim

**Affiliations:** 1 Department of Internal Medicine, Seoul National University Hospital, Seoul, Republic of Korea; 2 International Healthcare Center, Seoul National University Hospital, Seoul, Republic of Korea; Kyung Hee University School of Medicine, REPUBLIC OF KOREA

## Abstract

**Background:**

Annual vaccination for influenza is globally recommended for some prioritized groups due to its high morbidity and mortality. Until 2019, South Korea has provided free influenza vaccination to children aged ≤12, adults aged ≥65, and pregnant women to enhance vaccination coverage. In 2020, with the COVID-19 pandemic, free flu vaccination was temporarily broadened to adults aged 62–64 and children aged 13–18. We analyzed the trends in influenza vaccination coverages in South Korea and evaluated the impact of the COVID-19 pandemic and the expansion of the free vaccination policy on influenza vaccination coverage.

**Methods:**

We conducted a cross-sectional study with nationwide survey data from Korea National Health and Nutrition Examination Survey (KNHANES). We evaluated the trends in influenza vaccination coverages of target populations from 2010 to 2020. Influenza vaccination coverages of children aged 13–18, adults aged 62–64, and adults aged ≥65 were compared between 2019 and 2020.

**Results:**

In total, 72,443 individuals were analyzed. From 2019 to 2020, with the expansion of free influenza vaccination and the COVID-19 pandemic, the vaccination coverage of children aged 13–18 increased from 27.8% to 43.5% (P<0.001) but that of people aged 62–64 showed insignificant change from 57.4% to 51.5% (P = 0.266). Furthermore, the vaccination coverage in adults aged ≥65 declined from 87.2% to 79.1% (P<0.001).

**Conclusion:**

In 2020, along with COVID-19 outbreaks, a decline of influenza vaccination coverage in older adults was observed regardless of free immunizations. It is likely due to behavioral changes to reduce the risk of COVID-19 transmission. This is supported by a greater reduction of influenza vaccination coverage in regions with higher COVID-19 outbreaks, as well as by South Korea’s high medical accessibility and highly congested medical facilities. To sustain a high level of vaccination coverage of high-risk population during epidemics, additional efforts beyond free vaccination policies should be implemented.

## Introduction

Influenza is a highly contagious acute respiratory infectious disease caused by influenza viruses that circulate annually [1]. Seasonal influenza is responsible for approximately 4,000,000 severe illnesses and 300,000 to 650,000 respiratory deaths globally [2–4]. One international meta-analysis showed that the estimated annual infection rate of influenza is 10% for unvaccinated children and 5% for unvaccinated adults, half of them being symptomatic infection [4]. In South Korea, the incidence of laboratory-confirmed influenza was 250 cases per 100,000 adults, influenza-associated admission was 58 cases per 100,000 adults, and influenza-associated death was 3 cases per 100,000 adults during the 2013–2014 season [5]. The socio-economic cost of influenza for adults was 125 million USD (138 billion KRW) during the same season in South Korea [5, 6]. Vaccination is a critical protection method for influenza infection. During 2010–2016, influenza vaccines prevented 1.6 million to 6.7 million influenza-related illnesses, 790,000 to 3.1 million medical visits, 39,000 to 87,000 hospitalizations, and 3,000 to 10,000 deaths in the United States annually [7].

Currently recommended prioritized groups for influenza vaccination were specified by the Centers for Disease Control and Prevention (CDC) in 2006 and have been modified according to the circumstances of each country [8]. In South Korea, the prioritized groups have been established in 2011 with reference to the recommendation and there has been no modification until now [9, 10]. Current prioritized groups for influenza vaccination designated by the CDC and Korea Disease Control and Prevention Agency (KDCA) are presented in Table 1. They include children aged 6–59 months, adults aged ≥50, pregnant women, people with chronic diseases, and immunosuppressed people.

**Table 1:** The prioritized groups for influenza vaccine designated by CDC and KDCA.

CDC	KDCA
High-risk groups for influenza complications	
· Children aged 6–59 months	· Children aged 6–59 months ^b^
· People aged ≥ 50	· The elderly aged ≥ 65 ^b^
	· People aged 50–64
· People with chronic diseases ^a^	· People with chronic diseases ^a^
· People who are immunosuppressed	· People who are immunosuppressed
· Pregnant women	· Pregnant women ^b^
· People aged 6 months– 18 years on long-term aspirin or salicylate-containing medications	· People aged 6 months– 18 years on long-term aspirin or salicylate-containing medications
· People who live in nursing homes and other long-term care facilities	· People who live in nursing homes and other long-term care facilities
· American Indian or Alaska Native persons	
· Obese people with BMI ≥40	
At risk of transmitting influenza to high-risk groups	
· Health care personnel	· Health care personnel
· Household contacts and caregivers of children aged < 5, adults aged ≥ 50, and people with medical conditions	· Household contacts and caregivers of infant aged < 6 months, adults aged ≥ 65, pregnant women, and people with medical conditions
People living in groups	
	· Children aged 5–18 years

^a^ Chronic pulmonary (including asthma), cardiovascular (excluding isolated hypertension), renal, hepatic, neurologic, hematologic, or metabolic disorders (including diabetes mellitus)

^b^ Individuals receiving free vaccinations under Korean National Immunization Program for Influenza

*CDC: Center for Disease Control and Prevention; KDCA: Korea Disease Control and Prevention Agency

In South Korea, the National Immunization Program (NIP) started in 1997 [11]. NIP provides various essential vaccinations to certain populations free of charge, and free influenza vaccinations are provided to some of the prioritized groups mentioned above. They were first put into action in 2005 for seniors aged ≥65 and have been expanded to children aged 6–59 months in 2017, children aged 6–12 in 2018, and pregnant women in 2019. The vaccination coverage in each group increased significantly after the change of policy [6, 12]. In 2020, with the COVID-19 pandemic, free flu vaccination was temporarily broadened to adults aged 62–64 and children aged 13–18 due to concerns about a simultaneous outbreak of influenza and COVID-19 [13].

The influenza vaccination program in South Korea is conducted by KDCA and municipal offices [10]. Vaccines in South Korea can be administered only at health care centers and consigned medical institutions under doctor’s prescription and supervision. Vaccine administration in pharmacies, workplaces, schools, or self-administration is not allowed.

The purpose of this study is to evaluate the effects and limitations of the expansion of the free vaccination policy for influenza vaccination in 2020 and evaluate the impact of the COVID-19 pandemic by comparing immunization coverages between 2019 and 2020. We evaluated the trend of influenza vaccination coverages in 2010–2020, especially focusing on the change between the pre- and post-COVID-19 years. In the end, we provided suggestions of policy to improve the current situation and prepare for similar future events.

## Material and methods

### Study design and population

We analyzed the data retrieved from the Korea National Health and Nutrition Examination Survey (KNHANES) conducted by Korea Disease Control and Prevention Agency (KDCA) from 2010 to 2020. KNHANES is an annual population-based cross-sectional survey to assess the health and nutritional state of the Korean population. It samples 25 households from each of 192 regions and visits each household to survey about 10,000 people every year. Each examination comprises health screening, health interview, and nutritional survey including extensive amounts of data such as clinical and biochemical information for morbidity, body measures, vaccination and health checkups, quality of life, physical activity and impairment, education and economic activity, oral hygiene, and nutrient intake status. It is conducted by professional investigation teams consisting of trained nurses, nutritionists, and public health scientists. Every subject filled out written informed consent [11, 14]. This study was conducted in accordance with the Declaration of Helsinki and was approved by the institutional review board of Seoul National University Hospital (IRB no. E-2207-105-1341).

We collected data from 4 data sets: KNHANES V (2010–2012), VI (2013–2015), VII (2016–2018), and VIII (2019–2020). Participants of KNHANES 2013 were excluded because influenza survey data were not available (N = 8,018). Also, participants who neglected the influenza survey question (N = 4,584) or those who answered “I do not know” (N = 2,403) were excluded. Finally, 72,443 participants were included out of 87,448 respondents. The response rate ranged from 71.1% to 77.5% [15].

### Measures

According to the KDCA, children aged ≤18, adults aged ≥62, and pregnant women have been eligible for free vaccination in 2020 [13]. Except for 2020, children aged ≤12, adults aged ≥65, and pregnant women have been eligible for free vaccination since 2019.

Four subgroups were selected as target groups in our previous studies: adults aged ≥65, children aged ≤12, pregnant women, and people aged 19–64 with chronic diseases [6, 12]. In the present study, considering the policy changes in 2020, we added two target subgroups: adults aged 62–64 and children aged 13–18.

Self-reported influenza vaccination status was obtained from health interviews by asking about the receipt of influenza vaccines in the past 12 months [16]. The vaccination coverage was calculated as the number of individuals who responded ‘Yes’ divided by the sum of individuals with ‘Yes’ and ‘No’. The data of ‘Unknown’ and ‘null’ were excluded.

Chronic diseases were divided into the following categories: malignancies (stomach, liver, colon, breast, cervical, lung, thyroid, and others), kidney diseases (chronic kidney disease and diabetes mellitus), heart diseases (coronary heart disease, angina, myocardial infarction, and stroke), lung diseases (bronchial asthma and tuberculosis), and liver diseases (chronic viral hepatitis and liver cirrhosis).

The following sociodemographic factors were examined: gender (male or female), area of residence (city or rural), level of education (≤9, 10–12, ≥13 years), and household incomes (in quartiles). Eat-out frequency and region subgroups (17 in total) were analyzed additionally.

### Statistical analysis

Bivariate associations of categorical variables were assessed using the chi-square test [17]. Time trend analyses of influenza vaccination coverage were done using linear-by-linear association. Associations between sociodemographic factors and vaccination coverages were evaluated using multivariable logistic regression [18]. Two-tailed P-values of <0.05 were considered statistically significant. P-value, P-for-trend, 95% confidence interval (CI), and adjusted odds ratio (aOR) were reported as indicated. The sample weights were adjusted accounting for selection probabilities, survey nonresponse, post-stratification, and trimming of extreme weights [16]. These data were analyzed using STATA® version 17 for Windows (StataCorp LLC, College Station, TX) and R Statistical Software version 4.1.2 for Windows (R Core Team).

## Results

### Demographic characteristics and influenza vaccination coverages

Table 2 summarizes the study population’s general characteristics and influenza vaccination coverages. The total number of participants was 72,443 (54.9% being women). The vaccination coverage in people aged ≥65 was the highest (82.1%), followed by children aged ≤12 (68.1%) and people aged 62–64 (52.9%). Vaccination coverages in age 13–18 (26.4%) and age 19–61 (26.3%) were similarly low.

**Table 2:** General Characteristics and Influenza vaccination coverages (n = 72,443).

			Total		Vaccinated		p-value
Sociodemographic factors			n	weighted %(95%CI)	n	weighted IVC%(95%CI)	
	Age groups	≤12	10,865	11.7 (11.4–12.1)	7,459	68.1 (66.7–69.5)	<0.001
	(years)	13–18	4,595	7.3 (7.0–7.6)	1,295	26.4 (24.7–28.1)	
		19–61	39,325	64.8 (64.3–65.3)	10,949	26.3 (25.7–26.9)	
		62–64	3,255	3.1 (3.0–3.2)	1,709	52.9 (50.6–55.1)	
		≥65	14,403	13.1 (12.7–13.5)	11,815	82.1 (81.2–82.9)	
	Gender	Men	32,690	50.0 (49.6–50.4)	14,104	35.6 (34.9–36.4)	<0.001
		Women	39,753	50.0 (49.6–50.4)	19,123	43.2 (42.5–44.0)	
	Region	City	58,991	84.3 (82.8–85.8)	26,284	38.6 (37.9–39.2)	<0.001
		Rural	13,452	15.7 (14.2–17.2)	6,943	44.3 (42.7–45.9)	
	Education	≤9	20,541	38.2 (37.6–38.7)	12,634	56.4 (55.5–57.3)	<0.001
	(years)	10–12	17,777	30.6 (30.1–31.2)	6,169	28.2 (27.3–29.1)	
		≥13	23,942	31.2 (30.6–31.9)	7,270	29.8 (28.9–30.7)	
	Income	1Q	17,606	25.1 (24.4–25.8)	7,957	38.1 (37.0–39.2)	<0.001
		2Q	18,142	25.2 (24.6–25.9)	8,249	38.4 (37.3–39.6)	
		3Q	18,169	24.9 (24.4–25.5)	8,405	40.2 (39.1–41.3)	
		4Q	18,110	24.7 (23.9–25.6)	8,419	41.1 (39.9–42.2)	
Not recommended groups of influenza vaccine							
	Age 19–64, no diseases, no pregnant		35,316	57.4 (56.9–57.9)	9,855	26.0 (25.4–26.7)	<0.001
Recommended groups of influenza vaccine			37,127	42.6 (42.1–43.1)	23,372	57.5 (56.6–58.3)	
	Age(years)	≤18	15,460	19.0 (18.6–19.4)	8,754	52.2 (50.8–53.5)	<0.001
		≥65	14,403	13.1 (12.7–13.5)	11,815	82.1 (81.2–82.9)	
	Current pregnant		268	0.4 (0.3–0.4)	112	38.4 (31.6–45.7)	
	Age 19–64 with diseases		6,996	10.2 (9.9–10.5)	2,691	35.8 (34.3–37.3)	
	Diseases type	Malignancies	1,152	15.2 (14.3–16.2)	485	41.5 (38.1–44.9)	<0.001
		Kidney diseases	1,965	27.0 (25.8–28.2)	829	39.3 (36.7–42.0)	
		Heart diseases	808	10.6 (9.7–11.5)	315	37.2 (33.4–41.2)	
		Lung diseases	2,387	37.1 (35.6–38.5)	847	32.6 (30.5–34.8)	
		Liver diseases	692	10.2 (9.3–11.1)	220	27.5 (23.8–31.5)	

Heart diseases: coronary heart disease, myocardial infarction, angina, stroke

Kidney diseases: chronic kidney disease, diabetes mellitus

Lung diseases: bronchial asthma, tuberculosis

Liver diseases: chronic viral hepatitis, liver cirrhosis

1Q-4Q; 1Q = lowest quartile, 4Q = highest quartile, aOR: adjusted odds ratio, 95% CI: 95% confidence interval

IVC = influenza vaccination coverage (proportions vaccinated)

p-values were obtained by chi-square test.

People living in the rural area constituted 15.7% of the population and their vaccination coverage was higher than that of people in the city (44.3% to 38.6%, p<0.001). Higher vaccination coverages were observed in the lowest education level (56.4%) and highest income groups (41.1%), p<0.001 for both. People who ate out ≥3 times a week accounted for 60.7% of the total population, and their vaccination coverage was lower than those who ate out ≤2 times a week. People in the non-target group (age 19–64, not pregnant with no chronic diseases) accounted for 57.4% of the total population and their vaccination coverage was 26.0%. Pregnant women and people aged 19–64 with chronic diseases comprised 0.4% and 10.2% of the total population and showed 38.4% and 35.8% of vaccination coverages, respectively.

### Trends of vaccination coverages among the target groups

**Fig 1:**
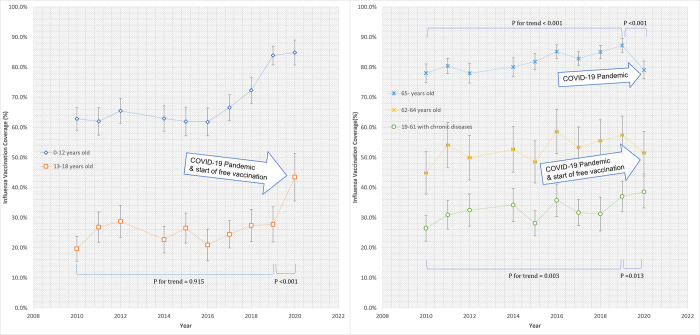
Trends of influenza vaccination coverages in target groups during 2010–2020.

Trends of influenza vaccination coverages in each target group over the 10 years are presented in Fig 1. The total vaccination coverage increased continuously from 33.3% in 2010 to 45.8% in 2019. The vaccination coverage in children aged ≤12 showed a dramatic increase after the implementation of free vaccination in 2017–2018, from 61.8% in 2016 to 83.9% in 2019 (p<0.001), and it sustained high in 2020. A similar steep increment of vaccination coverage was found in the children aged 13–18 between 2019 and 2020, from 27.8% to 43.5% (p<0.001), with the start of free vaccination and the COVID-19 pandemic. However, the vaccination coverage in the adults aged ≥65 declined from 87.2% to 79.1% (p<0.001), and the coverage in the adults aged 62–64 showed insignificant change, from 57.4% to 51.5% (p = 0.226) after the COVID-19 pandemic. People aged 19–61 with chronic diseases showed a gentle increase in the coverage from 26.5% in 2010 to 37.1% in 2019 (p-for-trend <0.001) and remained at the similar coverage in the pandemic year (38.5%, p = 0.013).

### The impact of Covid-19 pandemic on influenza vaccination coverage coverages

Table 3 compares influenza vaccination coverages for different sociodemographic factors and target subgroups between 2019 and 2020, which are pre- and post-COVID-19, respectively. Among different age groups, two groups showed statistically significant change in vaccination coverages: age 13–18 (27.8% to 43.5%, p < 0.001) and age ≥65 (87.2% to 79.1%, p < 0.001). Other age groups and sociodemographic factors (gender, region, education level, income quartile, and eat-out frequency) showed statistically insignificant temporal changes in vaccination coverages. The non-target group did not show a significant difference. People aged 19–64 with chronic diseases showed no significant temporal difference in total, but among them, the heart disease group alone showed a significant decrease in vaccination coverage (45.4% to 22.5%, p = 0.013). Pregnant women presented statistically insignificant decline in vaccination coverage, but it might be related to small sample size (N = 27 in 2019, N = 10 in 2020; 59.8% to 21.5%, p = 0.353).

**Table 3:** Comparison of influenza vaccination coverages between pre- and post-Covid-19 pandemic.

			Pre-Covid-19 (2019)	Post-Covid-19 (2020)	p-value
Sociodemographic factors			weighted IVC (95%CI)	weighted IVC (95%CI)	
	Age groups	≤12	83.9 (80.4–86.8)	84.9 (80.2–88.6)	0.985
	(years)	13–18	27.8 (22.2–34.1)	43.5 (35.6–51.7)	<0.001*
		19–61	30.8 (28.6–33.0)	32.1 (29.7–34.5)	0.448
		62–64	57.4 (50.9–63.7)	51.5 (44.4–58.6)	0.226
		≥65	87.2 (84.7–89.4)	79.1 (75.9–82.0)	<0.001*
	Gender	Men	42.4 (39.7–45.1)	42.4 (39.7–45.2)	0.985
		Women	49.3 (46.9–51.7)	49.3 (46.7–52.0)	0.975
	Region	City	44.9 (42.6–47.3)	45.5 (43.1–48.0)	0.739
		Rural	51.1 (46.7–55.4)	48.2 (44.0–52.3)	0.063
	Education	≤9	67.9 (65.3–70.5)	68.8 (65.5–71.9)	0.260
	(years)	10–12	33.5 (30.8–36.4)	35.1 (31.7–38.7)	0.124
		≥13	35.8 (32.9–38.9)	35.2 (32.6–37.9)	0.919
	Income	1Q	46.9 (43.5–50.3)	42.6 (39.1–46.2)	0.099
		2Q	42.4 (38.8–46.0)	43.5 (39.3–47.7)	0.700
		3Q	46.9 (43.6–50.2)	49.0 (44.8–53.2)	0.440
		4Q	47.5 (43.7–51.3)	48.1 (44.1–52.2)	0.818
Not recommended groups of influenza vaccine					
	Age 19–64, no diseases, no pregnant		30.3 (28.1–32.6)	31.7 (29.4–34.1)	0.410
Recommended groups of influenza vaccine			66.5 (63.6–69.3)	64.9 (61.8–67.9)	0.443
	Age(years)	≤12	83.9 (80.4–86.8)	84.9 (80.2–88.6)	0.985
		13–18	27.8 (22.2–34.1)	43.5 (35.6–51.7)	<0.001*
		≥65	87.2 (84.7–89.4)	79.1 (75.9–82.0)	<0.001*
	Current pregnant		59.8 (36.7–79.3)	21.5 (5.2–57.8)	0.353
	Age 18–64 with diseases		41.6 (36.8–46.7)	40.5 (35.5–45.8)	0.762
	Diseases type	Malignancies	49.2 (39.9–58.6)	43.8 (34.0–54.2)	0.925
		Kidney diseases	44.1 (36.5–51.9)	40.2 (32.8–48.1)	0.480
		Heart diseases	45.4 (33.7–57.6)	22.5 (12.6–37.0)	0.013*
		Lung diseases	37.3 (31.1–44.0)	42.4 (35.4–49.7)	0.308
		Liver diseases	36.7 (24.3–51.1)	34.4 (19.0–53.8)	0.492

Heart diseases: coronary heart disease, myocardial infarction, angina, stroke

Kidney diseases: chronic kidney disease, diabetes mellitus

Lung diseases: bronchial asthma, tuberculosis

Liver diseases: chronic viral hepatitis, liver cirrhosis

1Q-4Q; 1Q = lowest quartile, 4Q = highest quartile, aOR: adjusted odds ratio, 95% CI: 95% confidence interval

IVC = influenza vaccination coverage (proportions vaccinated)

p-values were obtained by chi-square test.

* p-values<0.05

Because people aged ≥65 were not subject to free vaccination policy alterations and the vaccination coverage of them alone decreased in 2020, they were analyzed separately to evaluate the associated factors related to the decline of the vaccination coverage, as presented in Table 4. In addition to previously described sociodemographic factors, eat-out frequency and region subgroup data were analyzed together. Among the 17 different region subgroups, ‘first outbreak regions’ comprised two regions (Daegu and Gyeongsangbuk-do) where the first COVID-19 outbreak occurred. Adjusted odds ratios were obtained by logistic regression in 2019 and 2020 separately. In 2019, age ≥75, the presence of chronic diseases, and the residence in first outbreak regions were related to high vaccination coverage. In 2020, however, these statistical significances disappeared, suggesting the decline of vaccination coverages in these groups. The number of COVID-19 cases per 100,000 people in 2020 in the first outbreak regions were 189.3 while those of the other regions were 82.8. A more detailed analysis of people aged ≥65 is presented in S1 Table. In addition, the number of COVID-19 cases per 100,000 people of 17 region subgroups are presented in S2 Table. Stay-at-home orders by the government were equally implemented in all regions in 2020, while those in Seoul, Incheon, and Gyeonggi-do were slightly more intensive [19].

**Table 4:** Comparison of influenza vaccination coverage between pre- and post-Covid-19 pandemic among 65 years or older.

				Pre-Covid-19 (2019)			Post-Covid-19 (2020)	
Sociodemographic factors			IVC(%)	aOR (95% CI)	p-value	IVC(%)	aOR (95% CI)	p-value
	Age groups	65–74	84.7	1		79.7	1	
	(years)	75-	89.7	1.52 (1.07–2.15)	0.019*	83.5	1.21 (0.87–1.69)	0.242
	Gender	Men	86.9	1		79.2	1	
		Women	86.5	0.88 (0.62–1.25)	0.473	82.6	1.08 (0.78–1.52)	0.614
	Region	City	87.5	1		81.2	1	
		Rural	84.6	0.72 (0.50–1.03)	0.076	81.0	0.93 (0.66–1.31)	0.680
	Education	≤9	87.6	1		82.2	1	
	(years)	10–12	85.0	0.70 (0.46–1.06)	0.098	80.9	0.98 (0.67–1.45)	0.936
		≥13	83.9	0.59 (0.35–1.00)	0.050	77.0	0.99 (0.60–1.64)	0.970
	Income	1Q	84.2	1		78.5	1.0	
		2Q	88.8	1.56 (0.98–2.50)	0.055	84.6	1.53 (1.00–2.34)	0.052
		3Q	86.4	1.30 (0.83–2.04)	0.246	84.3	1.42 (0.92–2.18)	0.116
		4Q	87.2	1.43 (0.90–2.29)	0.133	77.7	1.13 (0.73–1.75)	0.579
Chronic Diseases		0	84.3	1		78.8	1	
		1+	89.4	1.54 (1.10–2.14)	0.011*	83.5	1.19 (0.88–1.61)	0.246
Eat-Out Frequency		≥3	85.7	1		76.1	1.0	
	(per week)	≤2	87.8	1.17 (0.79–1.75)	0.432	81.3	1.34 (0.89–2.02)	0.167
Region subgroups		Others	86.0	1.0	0.023*	81.1	1	0.766
		The first outbreak	91.0	2.00 (1.10–3.62)		81.5	1.08 (0.66–1.77)	
Total			86.7			81.1		

1Q-4Q; 1Q = lowest quartile, 4Q = highest quartile, aOR: adjusted odds ratio, 95% CI: 95% confidence interval

IVC = influenza vaccination coverage (proportions vaccinated)

P-values and aOR were obtained by logistic regression adjusted by sex, age, residual area, education level, house monthly income, chronic diseases, eat-out frequency, and region subgroups

* p-values<0.05

The first outbreak areas in South Korea: Daegu, Gyeongsangbuk-do

The number of confirmed COVID-19 cases in the first outbreak area was 189 per 100,000 people, and 84 in other areas.

## Discussion

In this study, we affirmed several factors previously reported to be related to high influenza vaccination coverages: the elderly and children, female gender, rural residence, low education level, and high income [6, 12]. Discrepancy in vaccination coverages among target groups was also present: relatively low coverages were observed in pregnant women and people with chronic diseases. Previous studies showed that financial support is one of the most powerful factors which enhance influenza vaccination coverages [6, 12, 20, 21]. As shown in Fig 1, vaccination coverages of children ≤12 increased steeply after 2017–2018 along with the start of free vaccination. With the outbreak of COVID-19 in 2020, however, the expansion of free vaccination resulted differently in two groups: children aged 13–18 showed a profound increase in the vaccination rate but adults aged 62–64 showed insignificant change. In addition, adults aged ≥65 showed declined vaccination coverage in 2020 although they have been receiving free vaccinations and have shown the highest coverages of all groups. In other words, older people received fewer influenza vaccinations after the COVID-19 outbreak. The discrepancy between the three age groups (13–18, 62–64, and ≥65) implies that the behavioral response to the COVID-19 pandemic was different according to age.

In South Korea, the first COVID-19 case was confirmed on January 20^th^, 2020, and nationwide stay-at-home orders were started on February 29^th^, 2020 [19]. There has not been a compulsory lockdown that might have prohibited people from getting vaccinated and there were no obstacles in terms of vaccine supplies [22]. So, the decline in vaccination coverage in older people is considered largely due to their voluntary behavioral changes. As respiratory diseases, influenza and COVID-19 share similar high-risk groups. However, their prevention method conflicted with each other: vaccination against influenza versus physical distancing against COVID-19. It was because there was no vaccine available for COVID-19 in South Korea until February 2021 and because in South Korea, people were only allowed to be vaccinated in doctor’s clinics, which exposes people to the risk of COVID-19 transmission. The fact that influenza vaccination coverages of older adults declined during the COVID-19 outbreaks suggests that COVID-19 was deemed more hazardous than influenza to older adults. Table 4 partly corroborates this assumption. The residency in first outbreak regions of older adults was no longer associated with higher influenza vaccination coverage after COVID-19. Also, similar patterns were observed in people aged ≥75 and people with chronic diseases who are under higher risks of severe COVID-19 infection. Both observations suggest that the potential risk of COVID-19 transmission during vaccination visits could have been the determinant factor.

In 2020, after the outbreak of COVID-19, numerous countries have implemented various government policies to increase flu vaccination coverage to prevent the co-pandemic of COVID-19 and influenza [23]. The policies included the expansion of funding and target population for influenza vaccination, the reinforcement of catch-up vaccination for unvaccinated people, allowing alternative vaccination sites, and the modification of the vaccination procedures not to compromise physical distancing (for example, pre-scheduled clinic visits, extending clinic hours, or outdoor vaccination) [23–32]. Thanks to these efforts, the majority of OECD member countries showed similar or increased influenza vaccination coverage of adults aged ≥65 during the pandemic [33, 34]. Detailed numbers are presented in S3 Table. However, vaccination coverage of adults aged ≥65 in South Korea, which was the highest of all countries in 2019, largely decreased after the COVID-19 outbreak.

There are several reasons why the decline was so prominent in South Korea unlike other countries. Influenza vaccination coverage of adults aged ≥65 in 2019 was 87.2% in South Korea and it was almost twice the average of OECD members. Full financial support to older adults since 2005 and high access to health care contributed to the high figure. However, this high medical accessibility–highest number of outpatient visits per person among the OECD members in 2020, being 2.5 times the average–is suspected to be the pitfall during the pandemic, because medical centers are usually crowded with walk-in patients, exposing the visitors for vaccinations to the risk of COVID-19 transmission, especially because there was no alternative route to get vaccinated in South Korea. In contrast, there were countries where vaccinations were available through pharmacies, stores, home doctors, or self-administration which could have been affected less. To cope with the pandemic, South Korea increased the total dose of vaccines by 25% and expanded social welfare facilities where visiting doctors can vaccinate the disabled, but it had insufficient effect.

It is highly recommended that influenza vaccination should be administered during the pandemic to prevent the co-infection of influenza and COVID-19 or other respiratory diseases [35, 36]. One study in Italy also showed that influenza vaccination coverages were positively correlated to favorable COVID-19 outcomes [37]. This indicates that more powerful systemic strategies along with free vaccination are required in South Korea and other countries with similar vaccination systems to enhance influenza vaccination coverage during the respiratory infectious disease epidemic such as COVID-19.

We suggest some tactics to keep the vaccination coverage in older adults high. First, the fear of overcrowded medical clinics should be resolved. Previously attempted policies such as monitoring entrances and exits, spacing queues, or scheduled vaccination visits could keep physical distancing but were not very effective to change people’s behavior. Alternative places for vaccination would be needed such as schools, workplaces, or outdoor vaccination booths with attending medical staffs. Homebound vaccination for high-risk people would also be effective. In addition, healthcare providers should suggest influenza vaccinations for people who visit hospitals for other reasons to minimize the number of clinic visits. Second, under the current situation that booster vaccines for COVID-19 are being recommended, co-inoculation of influenza and COVID-19 vaccine can be successful. Several clinical trials have recently investigated the co-inoculation of COVID-19 vaccines with seasonal influenza vaccines, revealing low safety concerns and sustained immunogenicity of both vaccines [38–40]. Furthermore, combination vaccines that contain both COVID-19 and influenza could reduce missed opportunities for vaccination and vaccine hesitancy [41, 42]. However, challenges exist such as interactions between the two compounds, dissimilar storage requirements, or uncertain seasonality of COVID-19 [43]. Several clinical trials are currently underway to combine COVID-19 vaccines with next-generation influenza vaccines, including modified mRNA-based influenza vaccines or Matrix-M-adjuvanted nanoparticle influenza vaccines [44, 45]. Third, a campaign that emphasizes the importance of influenza vaccination in the epidemic should be provided thoroughly along with other strategies. People can be easily influenced by beliefs such as the potential harm of getting two vaccinations at the same time. The government, media, and healthcare providers should make maximal efforts to inform the importance of influenza vaccination.

There are several limitations in this study. First, people’s vaccination behavior is largely contributed to cultural background, medical infrastructure, or government policies, so it is difficult to apply this result globally. Second, we examined the KNHANES data up to 2020, because the data of 2021 has not been reported yet. There were some major differences between 2020 and 2021, such as the implementation of COVID-19 vaccines and a change in the free vaccination policy (children aged 13–18 and adults aged 62–64 were excluded again). This study could not examine this data altogether. Third, small numbers of respondents in some target subgroups led to the lack of statistical power. Follow-up analysis with the accumulated data of the next several years is warranted.

## Conclusions

Free vaccination policies for various population have enhanced influenza vaccination coverage in South Korea. However, in 2020 along with COVID-19 outbreaks, a decline of influenza vaccination coverage in older adults was observed regardless of free immunizations. It is likely due to behavioral changes to reduce the risk of COVID-19 transmission. This is supported by a greater reduction of influenza vaccination coverage in regions with higher COVID-19 outbreaks, as well as by South Korea’s high medical accessibility and highly congested medical facilities. To sustain a high level of vaccination coverage among the people at high risk for influenza during epidemics, additional efforts beyond free vaccination policies should be implemented.

## Supporting information

S1 Table Comparison of influenza vaccination coverage between pre- and post-Covid-19 pandemic years among 65 years or older (detailed). (PDF)

S2 Table Number of COVID-19 cases per 100,000 people by each region. (PDF)

S3 Table Influenza vaccination coverages of OECD member countries for population aged 65 and over, 2019 and 2020. (PDF)

S1 Dataset Minimized dataset used in the study. (DTA)

S1 File Codebook for S1 Dataset. (DOCX)
