# Supplementary material for: The impact of the COVID-19 pandemic and the expansion of free vaccination policy on influenza vaccination coverage: An analysis of vaccination behavior in South Korea
Source: PLoS One. 2023 Feb 15;18(2):e0281812. doi: 10.1371/journal.pone.0281812 (PMC9931130; doi:10.1371/journal.pone.0281812)
Supplement: S1 Table — (PDF) [file pone.0281812.s001.pdf]

**S1 Table.** Comparison of Influenza vaccination coverage between pre- and post-Covid-19 pandemic years among 65 years or older (detailed).

| | | Vaccinated | | | | | | | | | | | |
|---|---|---|---|---|---|---|---|---|---|---|---|---|---|
| | | Pre Covid-19 (2019) | | | | | Post Covid-19 (2020) | | | | | Delta(Δ) | p-value |
| Sociodemographic factors | | Total | Vaccinated | IVC(%) | aOR (95% CI) | p-value | Total | Vaccinated | IVC(%) | aOR (95% CI) | p-value | | |
| Age groups | 65-74 | 928 | 786 | 84.7 | 1 | | 877 | 699 | 79.7 | 1 | | -5.0 | **0.005*** |
| (years) | 75- | 603 | 541 | 89.7 | 1.4 (0.9-2.2) | 0.113 | 510 | 426 | 83.5 | 1.4 (0.9-2.1) | 0.111 | -6.2 | **0.002*** |
| Gender | Men | 663 | 576 | 86.9 | 1 | | 615 | 487 | 79.2 | 1 | | -7.7 | **<0.001*** |
| | Women | 868 | 751 | 86.5 | 1.0 (0.7-1.5) | 0.991 | 772 | 638 | 82.6 | 1.1 (0.7-1.6) | 0.703 | -3.9 | **0.029*** |
| Region | City | 1097 | 960 | 87.5 | 1 | | 1003 | 814 | 81.2 | 1 | | -6.4 | **<0.001*** |
| | Rural | 434 | 367 | 84.6 | 0.5 (0.3-1.0) | **0.045*** | 384 | 311 | 81.0 | 1.1 (0.7-1.6) | 0.746 | -3.6 | 0.176 |
| Education | ≤9 | 1046 | 916 | 87.6 | 1 | | 901 | 741 | 82.2 | 1 | | -5.3 | **0.001*** |
| (years) | 10-12 | 300 | 255 | 85.0 | 0.6 (0.4-1.1) | 0.095 | 299 | 242 | 80.9 | 1.0 (0.6-1.7) | 0.930 | -4.1 | 0.186 |
| | ≥13 | 180 | 151 | 83.9 | 0.7 (0.4-1.4) | 0.333 | 174 | 134 | 77.0 | 1.4 (0.8-2.7) | 0.274 | -6.9 | 0.102 |
| Income | 1Q | 368 | 310 | 84.2 | 1 | | 330 | 259 | 78.5 | 1.0 | | -5.8 | **0.051*** |
| | 2Q | 392 | 348 | 88.8 | 1.8 (1.0-3.1) | 0.052 | 345 | 292 | 84.6 | 1.4 (0.8-2.2) | 0.233 | -4.1 | 0.097 |
| | 3Q | 383 | 331 | 86.4 | 1.6 (0.9-2.8) | 0.096 | 356 | 300 | 84.3 | 1.2 (0.7-2.0) | 0.438 | -2.2 | 0.408 |
| | 4Q | 382 | 333 | 87.2 | 1.5 (0.9-2.7) | 0.130 | 346 | 269 | 77.7 | 1.0 (0.6-1.7) | 0.882 | -9.4 | **0.001*** |
| Chronic Diseases | 0 | 807 | 680 | 84.3 | 1 | | 709 | 559 | 78.8 | 1 | | -5.4 | **0.006*** |
| | 1+ | 724 | 647 | 89.4 | 1.8 (1.2-2.7) | **0.004*** | 678 | 566 | 83.5 | 1.3 (0.9-1.8) | 0.180 | -5.9 | **0.001*** |
| Eat Out Frequency | ≥3 | 272 | 233 | 85.7 | 1 | | 184 | 140 | 76.1 | 1.0 | | -9.6 | **0.009*** |
| (per week) | ≤2 | 1132 | 994 | 87.8 | 1.1 (0.7-1.8) | 0.610 | 937 | 762 | 81.3 | 1.5 (1.0-2.2) | **0.047*** | -6.5 | **<0.001*** |
| Region subgroups | Seoul | 280 | 233 | 83.2 | 1 | | 247 | 206 | 83.4 | 1 | | 0.2 | 0.954 |
| | Busan | 100 | 87 | 87.0 | 1.2 (0.7-2.3) | 0.505 | 82 | 64 | 78.0 | 0.8 (0.3-1.9) | 0.425 | -9.0 | 0.110 |
| | **Daegu** | 113 | 105 | 92.9 | 2.9 (1.0-8.2) | **0.044*** | 53 | 39 | 73.6 | 0.6 (0.2-1.7) | 0.154 | -19.3 | **0.001*** |
| | Incheon | 67 | 57 | 85.1 | 1.4 (0.7-3.0) | 0.343 | 47 | 42 | 89.4 | 3.4 (0.8-15.0) | 0.113 | 4.3 | 0.505 |
| | Gwangju | 44 | 37 | 84.1 | 1.3 (0.3-5.0) | 0.692 | 55 | 44 | 80.0 | 0.5 (0.2-1.4) | 0.276 | -4.1 | 0.600 |
| | Daejeon | 56 | 50 | 89.3 | 1.5 (0.5-4.2) | 0.448 | 63 | 51 | 81.0 | 1.4 (0.6-3.3) | 0.760 | -8.3 | 0.205 |
| | Ulsan | 15 | 11 | 73.3 | 0.4 (0.1-1.5) | 0.164 | 23 | 19 | 82.6 | 0.9 (0.3-2.9) | 0.707 | 9.3 | 0.493 |
| | Sejong | 16 | 15 | 93.8 | N/A | N/A | 30 | 24 | 80.0 | 1.1 (0.2-6.5) | 0.810 | -13.8 | 0.216 |
| | **Gyeonggi-do** | 271 | 237 | 87.5 | 2.0 (1.1-3.7) | **0.034*** | 263 | 211 | 80.2 | 0.9 (0.4-1.7) | 0.334 | -7.2 | **0.023*** |
| | Gangwon-do | 54 | 45 | 83.3 | 1.2 (0.6-2.5) | 0.593 | 90 | 77 | 85.6 | 0.9 (0.3-2.6) | 0.451 | 2.2 | 0.720 |
| | ChungCheongbuk-do | 73 | 64 | 87.7 | 2.7 (1.4-5.4) | **0.004*** | 45 | 33 | 73.3 | 0.5 (0.2-1.0) | **0.021*** | -14.3 | **0.048*** |
| | Chungcheongnam-do | 65 | 52 | 80.0 | 1.3 (0.4-4.1) | 0.707 | 29 | 23 | 79.3 | 0.8 (0.4-1.9) | 0.298 | -0.7 | 0.939 |
| | Jeollabuk-do | 64 | 52 | 81.3 | 1.6 (0.6-4.0) | 0.307 | 64 | 54 | 84.4 | 1.1 (0.6-1.9) | 0.604 | 3.1 | 0.639 |
| | Jeollanam-do | 82 | 72 | 87.8 | 1.8 (0.3-12.9) | 0.544 | 61 | 47 | 77.0 | 0.5 (0.3-1.1) | 0.158 | -10.8 | 0.089 |
| | Gyeongsangbuk-do | 86 | 76 | 88.4 | 4.0 (1.1-15.3) | **0.042** | 98 | 84 | 85.7 | 1.3 (0.5-3.1) | 0.802 | -2.7 | 0.593 |
| | Gyeongsangnam-do | 113 | 104 | 92.0 | 3.9 (1.4-10.7) | **0.008** | 104 | 79 | 76.0 | 0.6 (0.3-1.4) | 0.380 | -16.1 | **<0.001*** |
| | Jeju-do | 32 | 30 | 93.8 | 5.0 (0.5-48.5) | 0.167 | 33 | 28 | 84.8 | 0.9 (0.2-3.6) | 0.825 | -8.9 | 0.247 |
| | Total | 1531 | 1327 | 86.7 | | | 1387 | 1125 | 81.1 | | | -5.6 | <0.001* |

1Q-4Q; 1Q = lowest quartile, 4Q = highest quartile, aOR: adjusted odds ratio, 95% CI: 95% confidence interval
IVC = influenza vaccination coverage (propotions vaccinated)
p-values and aOR were obtained by logistic regression adjusted by sex, age, residual area, education level, house monthly income, and region subgroups
*** p-values<0.05**
