# Supplementary material for: The impact of the COVID-19 pandemic and the expansion of free vaccination policy on influenza vaccination coverage: An analysis of vaccination behavior in South Korea
Source: PLoS One. 2023 Feb 15;18(2):e0281812. doi: 10.1371/journal.pone.0281812 (PMC9931130; doi:10.1371/journal.pone.0281812)
Supplement: S2 Table — (PDF) [file pone.0281812.s002.pdf]

S2 Table. Number of COVID-19 cases per 100,000 people by each region.

	Region	COVID-19 cases	Total population	Cases per 100,000
1	Seoul	15,427	9,668,465	160
2	Busan	1532	3,391,946	45
3	**Daegu***	7504	2,418,346	**310**
4	Incheon	2330	2,942,828	79
5	Gwangju	863	1,450,062	60
6	Daejeon	710	1,463,882	49
7	Ulsan	531	1,136,017	47
8	Sejong	109	355,831	31
9	Gyeonggi-do	11,683	13,427,014	87
10	Gangwon-do	989	1,542,840	64
11	ChungCheongbuk-do	868	1,600,837	54
12	Chungcheongnam-do	1237	2,121,029	58
13	Jeollabuk-do	649	1,804,104	36
14	Jeollanam-do	471	1,851,549	25
15	**Gyeongsangbuk-do***	2071	2,639,422	**78**
16	Gyeongsangnam-do	1034	3,340,216	31
17	Jeju-do	302	674,635	45
Total		48,312	51,829,023	93

* The first outbreak areas in South Korea
