# Supplementary material for: The impact of the COVID-19 pandemic and the expansion of free vaccination policy on influenza vaccination coverage: An analysis of vaccination behavior in South Korea
Source: PLoS One. 2023 Feb 15;18(2):e0281812. doi: 10.1371/journal.pone.0281812 (PMC9931130; doi:10.1371/journal.pone.0281812)
Supplement: S3 Table — (PDF) [file pone.0281812.s003.pdf]

Table S3. Influenza vaccination coverages of OECD member countries for population aged 65 and over, 2019 and 2020.

	Country	2019	2020
1	Australia	56.2	62.2
2	Austria	18.3	N/A
3	Canada	60.2	64.7
4	Chile	68.3	84.7
5	Costa Rica	64	71
6	Czech Republic	22.9	23.9
7	Denmark	52	75
8	Estonia	15	12
9	Finland	47.3	53.7
10	France	52	59.9
11	Germany	38.8	47.3
12	Greece	58.9	73.5
13	Hungary	23.6	30.5
14	Iceland	47.5	54.6
15	Ireland	58.9	70.5
16	Israel	59.8	68.4
17	Italy	53.1	54.6
18	Japan	50	66
19	**South Korea**	**85.8**	**80.7**
20	Latvia	11.7	9.1
21	Lithuania	21.5	26.3
22	Luxembourg	40.4	46.3
23	Mexico	76.1	72.6
24	Netherlands	61.3	67.9
25	New Zealand	62	73
26	Norway	43	57
27	Poland	10.4	N/A
28	Slovak Republic	11.5	12.8
29	Slovenia	18.8	27
30	Spain	54.7	67.7
31	Sweden	52.8	60.4
32	Türkiye	5.9	N/A
33	United Kingdom	72.4	72.4
34	United States	67	67.5
