# Supplementary material for: The impact of the COVID-19 pandemic and the expansion of free vaccination policy on influenza vaccination coverage: An analysis of vaccination behavior in South Korea
Source: PLoS One. 2023 Feb 15;18(2):e0281812. doi: 10.1371/journal.pone.0281812 (PMC9931130; doi:10.1371/journal.pone.0281812)
Supplement: S1 File — (DOCX) [file pone.0281812.s005.docx]

CODE BOOK

| Name of Variables | Labels |
| --- | --- |
| year | Survey year |
| wt_itvex | Survey weight |
| kstrata | Survey strata |
| age | Age(year) |
| sex | Gender |
| town_t | Residual area |
| region | Region subgroup |
| ainc | Home income per month |
| edu | Education level |
| l_out_fq | Frequency of eat-out |
| bh9_11 | Self-reported influenza vaccination status |
| he_prg | Current pregnant |
| di3_dg | Stroke |
| di4_dg | Coronary heart disease |
| di5_dg | Myocardial infarction |
| di6_dg | Angina |
| dj2_dg | Tuberculosis |
| dj4_dg | Bronchial asthma |
| dk4_dg | Liver cirrhosis |
| dk8_dg | Chronic viral hepatitis, B |
| dk9_dg | Chronic viral hepatitis, C |
| dn1_dg | Chronic kidney disease |
| de1_dg | Diabetes mellitus |
| ca | Malignancies |
